# Persistence and Determinants of Late‐Life Depression: Results of the Nationally Representative Longitudinal German Aging Survey 2008 to 2023

**DOI:** 10.1002/gps.70181

**Published:** 2025-12-17

**Authors:** M. W. Stratmann, H.‐H. König, A. Hajek

**Affiliations:** ^1^ Department of Health Economics and Health Services Research University Medical Center Hamburg‐Eppendorf Hamburg Germany

**Keywords:** elderly, incidence, late‐life depression, mental health, old age, persistent depressive disorder, prevalence, risk factor

## Abstract

**Objectives:**

Late‐life depression is the most common mental disorder in older adults. As symptom progression and risk factors are not well understood, this study focuses on longitudinal symptom progression and time‐varying risk factors.

**Methods:**

Longitudinal data of adults aged 60+ were drawn from six waves (2008–2023) of the German Aging Survey (DEAS), with the largest analytic sample in 2014 (*n* = 6515). At each wave participants were classified as having “no” (0–9), “minor” (10–17) or “major” (18–45) depressive symptoms (DS) based on the German Center for Epidemiologic Studies Depression Scale (CES‐D). Persistent DS were defined as PDS (at least minor DS) or majorPDS (major DS) across two consecutive waves. Weighted transition probabilities between DS stages were calculated across all person‐years. Linear fixed effects regression with cluster‐robust standard errors was used to identify time‐varying determinants of DS.

**Results:**

In 2014, the prevalence rate was 17.4% [16.6%–18.1%] for minor DS, 7.2% [6.6%–7.8%] for major DS, 12.2% [11.3%–13.1%] for PDS and 2.5% [2.0%–3.1%] for majorPDS. Estimates were similar across all six waves. Minor DS frequently persisted (37.3% [34.1%–40.6%]) or progressed to major DS (11.7% [9.6%–14.2%]). Major DS persisted in 38.6% [31.1%–46.7%] and remitted to minor DS in 31.8% [25.9%–38.3%] of cases. Transition to widowhood (*β* = 0.67, *p* < 0.05), worsening of physical functioning (*β* = −0.06, *p* < 0.01), increasing loneliness (*β* = 1.02, *p* < 0.01), worsening of sleep quality (*β* = 0.78–4.40, *p* < 0.01) and decreasing BMI (*β* = −0.08, *p* < 0.01) were associated with increases in DS.

**Conclusions:**

DS are common in later life and are frequently persistent. Minor DS often persist or worsen, underscoring their role as a key risk factor. Major DS frequently remits only partly. Early, targeted interventions could be informed by modifiable determinants that may also help to allocate scarce mental health resources effectively.

## Highlights

- About 17% and 7% of German older adults report minor and major depressive symptoms respectively
- Minor depressive symptoms will persist or worsen in about half of cases
- Major depressive symptoms persist in about 40% of cases
- Time‐varying risk factors were analyzed using longitudinal data spanning 15 years

## 1 Introduction

Depression is the most common mental disorder in late life [1, 2], with severe consequences and substantial burden for both the individual and society [3]. It is, among other things, associated with frailty [4], accelerated aging [5], and increased morbidity [6]. Globally, an estimated 7.2% of adults aged 60+ meet criteria for major depression [7], with a prevalence of around 5.0% among Germans aged 65+ [2], and higher rates in those aged 75+ [8].

Depression often presents differently in older adults [9, 10], with less affective symptoms (e.g., sadness) and more somatic symptoms (e.g., fatigue, appetite changes), cognitive changes and anhedonia [11]. Anhedonia, social withdrawal and cognitive slowing can be mistaken as normal aging or signs of dementia. And even though many older adults may not meet full diagnostic criteria for major depression, they are still experiencing substantial symptoms [10]. As a result, geriatric psychiatry has increasingly focused on minor (or subthreshold) depression ‐ a strong predictor of major depression [12] ‐ that also reduces quality of life and strains health care systems [13] due to its high prevalence.

While about one‐third of major depression cases in the general population become chronic, this rate may reach up to 50% or more in older adults [14, 15, 16]. Persistent depressive disorder (PDD), defined as depressive symptoms lasting two or more years [17], is associated with poorer outcomes than episodic depression [18, 19], yet has received little attention in late life [20]. Prevalence estimates vary widely [21, 22] and German population‐based data are limited to dysthymia, a milder persistent form: 2.5% in adults 65+ [2] and 5.1% in those 80+ [23], with a 2.2% annual incidence in those 65+ [24].

Numerous biological, psychological and social factors are linked to late‐life depression [11, 25]. While some, such as gender, education and genetics are time‐constant, others–like social isolation, poor physical health and limited mobility [26, 27]–are time‐varying and potentially modifiable, offering targets for secondary prevention and compensation strategies in aging populations.

Understanding prevalence, persistence and determinants of late‐life depression is essential to developing informed public health interventions. Identifying high‐risk groups can help allocate scarce resources, improve outcomes, and reduce healthcare burden. Only a few international studies have examined late‐life depression in a longitudinal context. There is a lack of studies based on German longitudinal data, despite its availability. For this reason, this study focuses on the course of late‐life depression, its persistence and time‐varying determinants of depressive symptoms (DS) in older adults in Germany. Approximate population‐based estimates were calculated, transitions between stages of DS were analyzed and associations of DS with time‐varying risk factors were explored.

## 2 Methods

This article follows the STROBE guidelines for observational studies [28].

### 2.1 Sample

Data were drawn from the German Aging Survey (DEAS), a nationally representative, longitudinal study of adults aged 40+ living in private households in Germany [29]. Funded by the Federal Ministry for Family Affairs, Senior Citizens, Women, and Youth in Germany (BMFSFJ) and conducted by the German Center of Gerontology (DZA), DEAS collects data on health, socioeconomic status, employment, family, and psychological well‐being [30]. Data has been collected in multiple waves since 1996, with measurement intervals of initially 6 years, and, since 2008, every 3 years. Refreshment samples were added 2002, 2008, and 2014. Refreshment was planned for 2020 but was prevented by the COVID‐19 pandemic. Random samples are based on a two‐stage sampling methodology: firstly, random sampling of 290 out of 12,000 municipalities as from 1996; secondly, private household samples based on the local population registries of these 290 municipalities [31]. Stratified selection according to age, gender and region (eastern/western Germany) took place, with oversampling of the oldest old, men and East Germans. Weights are computed and provided to adjust according to the national micro census data. The response rate of the latest refreshment sample in 2014 was 27.1% (*N* = 6002) [31], which is comparable to other high‐quality German health surveys [2, 32, 33]. Follow‐up rates are approximately 65% [34], indicating good adherence. Data was collected in computer‐assisted personal interviews (CAPI) or telephone interviews (CATI) for mostly sociodemographic information, and were followed by a written drop‐off questionnaire for more sensitive information (e.g., loneliness, life satisfaction) [34]. This study includes participants aged 60+ from six waves collected at 3‐year intervals: 2008, 2011, 2014, 2017, 2020/21 and 2023. Only waves with a 3‐year interval were selected to ensure uniform intervals. All participants provided written informed consent. Ethical approval was not required, as criteria such as medical risk or invasive procedure, were not met. Please see Klaus, Engstler [31] for further details.

### 2.2 Outcome: Depression

DS were assessed using the 15‐item German version of the Center for Epidemiologic Studies Depression Scale (CES‐D) [35, 36], which measures symptom frequency over the past week. Answers are coded as having values from 0 “rarely or not at all (less than 1 day)” to 3 “most of the time or all of the time (5–7 days)”. This results in a score ranging from 0 to 45. Studies have shown that the CES‐D is suitable for assessing depression in later life [37, 38]. A score of 18 or higher indicates clinically significant DS with sensitivity and specificity both above 80% for major depression [39]. Following prior research [40], participants were categorized as:

– no DS (0–9)
– minor DS (10–17)
– major DS (18–45).

Operationalization of prevalence and incidence is based on comparable studies [41, 42]. Prevalence was calculated as point prevalence of minor or major DS within each wave. Incidence was calculated as the occurrence of a new case of DS in a given wave among participants who did not meet criteria in the previous wave. Incidence of major DS reflects a score ≥ 18 in the current wave and < 18 in the previous wave; incidence of minor DS reflects a score of ≥ 10 and < 18 in the current wave and < 10 in the previous wave. Transitions between DS stages are analyzed later (see transition probabilities in 2.5).

Persistent DS (PDS) was calculated as repeated DS across two consecutive waves:

– PDS (score ≥ 10 in both waves)
– majorPDS (score ≥ 18 in both).

Of note, PDS represents a comprehensive category that includes cases with any DS, including majorPDS cases. The two categories are therefore not exclusive, but this operationalization of PDS follows the comprehensive DSM‐V definition of PDD, which encompasses mild, severe and fluctuating forms of depression [17].

Incident PDS was calculated as newly emerging PDS across the study's time period. For example, an incident case of majorPDS in 2014 would be a respondent who reported no major DS in 2008, but major DS in both 2011 and 2014. Similarly, an incident case of PDS in 2014 would be a respondent who reported no DS in 2008, but at least minor DS in both 2011 and 2014.

### 2.3 Sociodemographic Determinants

Participants reported their age in years and were categorized as younger old (60–69), middle old (70–79) and oldest old (80+). Categorization of education followed the International Standard Classification of Education (ISCED‐97): low (0–2), medium (3–4) and high (5–6) [43]. Gender (“men” or “women”) and marital status (“married”, “married but living separately”, “divorced”, “widowed” and “single”) were also reported.

### 2.4 Time‐Varying Social and Health‐Related Determinants

The number of self‐reported physical illnesses is based on a list of widespread disease (e.g., diabetes, cardiac and circulatory disorders) and ranges from 0 to 11. Physical functioning was measured using the subscale “Physical functioning” of the Short Form Health Survey 36 (SF‐36) [44, 45]. Participants reported how physically impaired they are in 10 daily activities (e.g., taking stairs, lifting groceries): “yes, limited a lot” (1), “yes, limited a little” (2) and “no, not limited at all” (3). The responses are transformed into functional levels of 0 (1), 50 (2), or 100 (3) points, so that the average of the 10 items has a range from 0 to 100, with higher values indicating better physical functioning. Loneliness was measured with the widely used De Jong Gierveld scale [46]. Participants responded on a 4‐point‐scale from “strongly agree” to “strongly disagree”. A mean score was calculated with higher values indicating higher loneliness (ranging from 1 to 4). Network size was reported indirectly. Participants were iteratively asked: “Which people are important to you? […] Please tell the name of the […] person with whom you have regular contact and who is important to you”. They further reported information on the relationship, gender, frequency of contact, and age of the contact person. Participants could name up to 8 people and were asked (if they reached the limit) how many more they would have named. Network size was based on the number of meaningful contacts with regular interactions reported and ranged from 0 to 9 and more (9+). Sleep quality was self‐reported as: “very good”, “good”, “fairly bad”, or “very bad”. The body‐mass‐index (BMI) was calculated as the ratio of body weight (in kg) and body height (in meters) squared. Physical activity was self‐reported as the amount of regular sport activity or exercises (“How often do you do sports such as hiking, soccer, gymnastics, or swimming?”): “daily”, “several times a week”, “1 to 3 times a month”, “less often”, or “never”.

### 2.5 Statistical Analysis

The prevalence and incidence of minor DS, major DS, PDS and majorPDS is reported as the absolute frequency, as well as the weighted percentages including 95% confidence intervals. Cross‐sectional and longitudinal weights from the DZA were applied [30]. Estimates are presented for the 2014 wave as this wave contained the largest analytic sample, has information on all indicators of interest, and thus provides the most precise estimates. Estimates for each wave are reported in the appendix. Only participants who had provided complete data on DS were included. Transition probabilities between DS stages are calculated using all available person‐years (2008–2023). This allowed for the analysis, for example, of the probability of persistence of major DS given the respondent's major DS in the previous wave.

To identify time‐varying determinants of metric DS (0–45), we applied linear fixed effects (FE) regression with cluster‐robust standard errors [47]. Longitudinal data allows for the separation of time‐constant and time‐varying factors [48]. FE regression isolates within‐person changes over time, controlling for all time‐constant determinants, observed (e.g., gender, education) or unobserved (e.g., family or genetic predisposition). This approach yields unbiased and consistent estimates for time‐varying predictors under relatively weak assumptions [49]. Our choice of FE over random‐effects was supported by Sargan‐Hansen tests (corresponds to Hausman‐tests with cluster‐robust standard errors). Due to the low proportion of missing values in the determinants examined, listwise deletion was used to address missing values. This approach yields comparable results when missing values are low, compared to more complex approaches such as multiple imputation in a longitudinal framework [50].

All analyses were conducted using StataNow 19.5 [51], with significance set at *p* < 0.05.

## 3 Results

### 3.1 Sample Characteristics

Characteristics of the 2014 sample (*N* = 6,515, aged 60+) are reported in Table 1, in total and by DS stage. Participants were on average 71.4 years old (SD = 7.2), with 47.3% of the sample being women. Most reported medium (51.1%) or high (39.4%) levels of education and were married and living with their partner (69.4%). Characteristics differed by DS stage and pairwise post‐hoc comparisons are presented in the Supporting Information S1: Table S1. Women, widowers, those with lower education level, more physical illnesses, poorer physical functioning, higher loneliness, poorer sleep quality and lower physical activity levels were among those participants with more severe DS.

**TABLE 1:** Sociodemographic information in 2014 in total and by depressive symptoms stage.

	DS stage				
	No DS	Minor DS	Major DS	Total	Test
*N*	4981 (76.5%)	1119 (17.2%)	415 (6.4%)	6515 (100.0%)	
Age	71.1 (7.1)	72.4 (7.7)	72.2 (7.7)	71.4 (7.2)	< 0.001
Age group					
60–69 years	2130 (42.8%)	407 (36.4%)	158 (38.1%)	2695 (41.4%)	< 0.001
70–79 years	2205 (44.3%)	495 (44.2%)	167 (40.2%)	2867 (44.0%)	
80+ years	646 (13.0%)	217 (19.4%)	90 (21.7%)	953 (14.6%)	
Gender					
Men	2764 (55.5%)	510 (45.6%)	159 (38.3%)	3433 (52.7%)	< 0.001
Women	2217 (44.5%)	609 (54.4%)	256 (61.7%)	3082 (47.3%)	
Level of education (ISCED)					
Low	392 (7.9%)	146 (13.0%)	84 (20.2%)	622 (9.6%)	< 0.001
Medium	2484 (49.9%)	613 (54.8%)	230 (55.4%)	3327 (51.1%)	
High	2103 (42.2%)	360 (32.2%)	101 (24.3%)	2564 (39.4%)	
Marital status					
Married	3572 (71.9%)	712 (63.8%)	224 (54.5%)	4508 (69.4%)	< 0.001
Married, living separately	59 (1.2%)	16 (1.4%)	11 (2.7%)	86 (1.3%)	
Divorced	420 (8.4%)	107 (9.6%)	41 (10.0%)	568 (8.7%)	
Widowed	736 (14.8%)	237 (21.2%)	111 (27.0%)	1084 (16.7%)	
Ingle	184 (3.7%)	44 (3.9%)	24 (5.8%)	252 (3.9%)	
Number of physical disease (self‐report) [0–11]	2.7 (1.8)	3.8 (2.0)	4.1 (2.1)	3.0 (1.9)	< 0.001
Physical functioning [0–100]	82.5 (20.6)	62.3 (28.9)	49.8 (32.5)	76.9 (25.4)	< 0.001
Loneliness [1–4]	1.7 (0.5)	2.0 (0.6)	2.2 (0.6)	1.8 (0.5)	< 0.001
Network size [0–9+]	5.0 (2.8)	4.6 (2.8)	4.3 (2.6)	4.9 (2.8)	< 0.001
Sleep quality					
Very good	1027 (25.7%)	83 (9.9%)	15 (5.2%)	1125 (22.0%)	< 0.001
Good	2282 (57.1%)	379 (45.1%)	90 (31.5%)	2751 (53.7%)	
Fairly bad	630 (15.8%)	317 (37.7%)	134 (46.9%)	1081 (21.1%)	
Very bad	58 (1.5%)	61 (7.3%)	47 (16.4%)	166 (3.2%)	
Body‐Mass‐Index	27.0 (4.3)	27.3 (4.8)	27.6 (5.3)	27.1 (4.5)	0.002
Physical activity					
Never	1463 (29.4%)	478 (42.7%)	225 (54.3%)	2166 (33.3%)	< 0.001
Less often	513 (10.3%)	114 (10.2%)	22 (5.3%)	649 (10.0%)	
1 to 3 times a month	321 (6.4%)	66 (5.9%)	12 (2.9%)	399 (6.1%)	
Once a week	856 (17.2%)	182 (16.3%)	55 (13.3%)	1093 (16.8%)	
Several times per week	1317 (26.5%)	202 (18.1%)	68 (16.4%)	1587 (24.4%)	
Daily	509 (10.2%)	77 (6.9%)	32 (7.7%)	618 (9.5%)	

*Note:* DS assessed using the 15‐item German version of the Center for Epidemiologic Studies Depression Scale (CES‐D): no DS (0–9), minor DS (10–17) and major DS (18–45). Physical functioning measured using the subscale “Physical functioning” of the Short Form Health Survey 36. Loneliness measured with the widely used De Jong Gierveld scale.

Abbreviations: DS, Depressive symptoms; ISCED, International Standard Classification of Education.

### 3.2 Prevalence and Incidence

Prevalence and incidence information for 2014 are reported in Table 2, in total and by gender. In total, 17.4% [16.6%–18.1%] met the criteria for minor DS and 7.2% [6.6%–7.8%] for major DS. Incidence was 9.3% [8.2%–10.5%] for minor DS and 4.4% [3.8%–5.0%] for major DS. PDS prevalence was 12.2% [11.3%–13.1%], majorPDS was 2.5% [2.0%–3.1%]. Corresponding incidence rates were 3.9% [3.4%–4.4%] and 0.8% [0.6%–1.0%].

**TABLE 2:** Prevalence and incidence information in 2014 in total and by gender.

	Gender					
	Men		Women		Total	
DS stage						
no DS	2764	80.5% [78.8%–82.2%]	2217	70.9% [68.3%–73.3%]	4981	75.4% [74.5%–76.3%]
minor DS	510	14.4% [12.9%–16.0%]	609	20.5% [18.3%–22.9%]	1119	17.4% [16.6%–18.1%]
major DS	159	5.1% [4.2%–6.2%]	256	8.7% [7.2%–0.3%]	415	7.2% [6.6%–7.8%]
Minor DS incidence	110	7.9% [6.4%–9.8%]	137	11.3% [9.1%–14.0%]	247	9.3% [8.2%–10.5%]
Major DS incidence	42	3.4% [2.3%–5.0%]	61	5.3% [3.6%–7.6%]	103	4.4% [3.8%–5.0%]
PDS	102	10.1% [7.9%–12.9%]	158	16.6% [12.7%–21.3%]	260	12.2% [11.3%–13.1%]
majorPDS	14	2.1% [1.1%–4.2%]	31	4.9% [2.4%–9.8%]	45	2.5% [2.0%–3.1%]
PDS incidence	37	3.3% [2.1%–5.1%]	60	6.9% [4.6%–10.0%]	97	3.9% [3.4%–4.4%]
majorPDS incidence	6	0.5% [0.2%–1.2%]	11	2.0% [0.7%–5.3%]	17	0.8% [0.6%–1.0%]

*Note:* DS assessed using the 15‐item German version of the Center for Epidemiologic Studies Depression Scale (CES‐D): no DS (0–9), minor DS (10–17) and major DS (18–45); minor DS incidence (score ≥ 10 and < 18 in current wave and < 10 in previous wave); major DS incidence (score ≥ 18 and < 18 in previous wave); PDS (score ≥ 10) and majorPDS (score ≥ 18) in two consecutive waves.

Abbreviations: DS, Depressive symptoms; PDS, Persistent depressive symptoms.

Women had higher prevalence rates than men for both minor DS (20.5% [18.3%–22.9%] vs. 14.4% [12.9%–16.0%]) and major DS (8.7% [7.2%–10.3%] vs. 5.1% [4.2%–6.2%]). This trend held for PDS and incidence, though confidence intervals overlapped. Prevalence and incidence information for 2014 by age group are reported in the Supporting Information S1: Table S2. Generally, estimates among the younger old (60–69) and middle old (70–79) were comparable, whilst significantly higher estimates were reported for the oldest old (80+). The highest incidence of PDS was found among oldest olds (15.4% [9.2%–24.6%]).

Supporting Information S1: Table S3 shows that prevalence and incidence rates are stable over time across waves from 2008 to 2023. Prevalence of minor DS varied from 16.9% [15.6%–18.1%] in 2008 to 18.1% [16.3%–20.1%] in 2017, major DS from 6.5% [5.7%–7.3%] in 2008 to 8.2% [6.7%–9.9%] in 2011. No differences were observed in waves 5 (2020) and 6 (2023), during and after the COVID‐19 pandemic.

### 3.3 Depression Trajectories

In Table 3 transition probabilities between DS stages are reported across all person‐years (*N* = 10,840). Among those with no DS, 83.6% [82.3%–84.8%] remained so, 12.8% [11.7%–14.0%] transitioned to minor DS, and 3.6% [3.0%–4.3%] to major DS. From minor DS, 51.0% [47.6%–54.4%] remitted, 37.3% [34.1%–40.6%] reported no change, and 11.7% [9.6%–14.2%] developed major DS. Of those with major, 38.6% [31.1%–46.7%] reported no change, 31.8% [25.9%–38.3%] remitted to minor DS and 29.6% [21.3%–39.5%] to no DS.

**TABLE 3:** Transitions between depressive symptoms stages.

	DS stage					
	No DS		Minor DS		Major DS	
Consecutive DS stage						
no DS	7418	83.6% [82.3%–84.8%]	876	51.0% [47.6%–54.4%]	158	29.6% [21.3%–39.5%]
minor DS	1022	12.8% [11.7%–14.0%]	590	37.3% [34.1%–40.6%]	189	31.8% [25.9%–38.3%]
major DS	248	3.6% [3.0%–4.3%]	182	11.7% [9.6%–14.2%]	157	38.6% [31.1%–46.7%]

*Note: N* = 10,840 person‐years. DS assessed using the 15‐item German version of the Center for Epidemiologic Studies Depression Scale (CES‐D): no DS (0–9), minor DS (10–17) and major DS (18–45).

Abbreviation: DS, Depressive symptoms.

Gender stratified transition probabilities are given in the Supporting Information S1: Tables S4 and S5. Confidence intervals overlap largely. However, reporting no DS across all waves, as well as remission from both minor DS and major DS is more likely for men than for women. Likewise, persistence of DS was higher among women than among men (major DS to major DS 27.1% [19.0%–37.2%] for men, 42.4% [32.7%–52.7%] for women).

Supporting Information S1: Tables S6–S8 report on age stratified transition probabilities. Transition from no DS to minor DS is more likely the older the age group (60–69: 9.4% [8.4%–10.6%]; 70–79: 13.3% [11.8%–15.0%]; 80+: 20.6% [16.2%–25.8%]). Confidence intervals for minor and major DS were large due to the small number of cases. Transitions (from no or minor DS) to major DS did not differ significantly. Similarly, remission rates from minor or major DS did not differ significantly.

### 3.4 Time‐Varying Determinants of Depressive Symptoms

Findings of linear FE regressions among the total sample are given in Table 4. Metric DS (0–45) were used as the outcome measure.

**TABLE 4:** Fixed effects regression.

	DS
Age	0.01
	(0.01)
Marital status (Reference: Married, living together)	
Married, living separately	0.44
	(0.56)
Divorced	−0.14
	(0.65)
Widowed	0.67*
	(0.26)
Single	0.00
	(1.10)
Number of physical disease (self‐report) [0–11]	0.04
	(0.03)
Physical functioning [0–100]	−0.06**
	(0.00)
Loneliness [1–4]	1.02**
	(0.14)
Network size [0–9+]	−0.01
	(0.02)
Sleep quality (Reference: Very good)	
Good	0.78**
	(0.11)
Fairly bad	2.67**
	(0.18)
Very bad	4.40**
	(0.39)
Body‐Mass‐Index	−0.08**
	(0.02)
Physical activity (Reference: Never)	
Less often	0.08
	(0.13)
1 to 3 times a month	−0.31
	(0.17)
Once a week	−0.20
	(0.13)
Several times per week	−0.42**
	(0.13)
Daily	−0.25
	(0.20)
Intercept	9.82**
	(1.04)
Number of observations	21,446
Number of groups	9107
*R*‐squared	0.11

*Note:* DS assessed using the 15‐item German version of the Center for Epidemiologic Studies Depression Scale (CES‐D). Physical functioning measured using the subscale “Physical functioning” of the Short Form Health Survey 36. Loneliness measured with the widely used De Jong Gierveld scale.

Abbreviation: DS, Depressive symptoms.

^**^ *p* < 0.01.

^*^ *p* < 0.05.

Increases in DS were not significantly associated with changes in age (*β* = 0.01, *p* > 0.05), number of physical diseases (*β* = 0.04, *p* > 0.05), and network size (*β* = −0.01, *p* > 0.05). With respect to marital status, transition to widowhood (*β* = 0.67, *p* < 0.05) compared to remaining “married” was significantly associated with increases in DS. Transitions to divorced (*β* = −0.14, *p* > 0.05) and single (*β* = 0.00, *p* > 0.05) were not. Increases in DS were further associated with decreases in physical functioning (*β* = −0.06, *p* < 0.01), increases in loneliness levels (*β* = 1.02, *p* < 0.01), decreases in sleep quality (changes from “very good” to “good”: *β* = 0.78, *p* < 0.01; to “fairly bad”: *β* = 2.67, *p* < 0.01); to “very bad”: (*β* = 4.40, *p* < 0.01) and decreases in BMI (*β* = −0.08, *p* < 0.01). For physical activity, only changes from “no activity” to “several times per week” yield significant results with decreases in DS (*β* = −0.42, *p* < 0.01). Linear FE regression results stratified by men and women are shown in the Supporting Information S1: Tables S9 and S10 and by age group in the Supporting Information S1: Tables S11–S13.

## 4 Discussion

This study examined longitudinal data on late‐life depression in Germany. Minor DS (17.4%), major DS (7.2%), at least minor PDS (12.2%) and majorPDS (2.5%) were common. Rates were highest for women and the oldest age group (80+). Estimates were stable across all six waves from 2008 to 2023. Of those with minor DS, over one‐third reported no change in symptoms, and one‐sixth progressed to major DS. Major DS persisted in about 40% of cases and remitted incompletely to minor DS in one third of cases. Regarding determinants, losing a spouse (i.e., becoming a widower), lower physical functioning, higher loneliness, poorer sleep quality, lower BMI and lower physical activity was associated with increases in DS. Our present study extends our current understanding of late‐life depression and especially persistent DS in older adults in Germany by analyzing longitudinal data spanning 15 years.

Overall, our estimates align with international cross‐sectional data: 17.4% for minor DS in Germany versus 18.6% globally for subthreshold depression using screening tools [52], and 7.2% for major DS exactly matching point estimate from international meta‐analysis based on categorical diagnosis [7]. Regarding PDS, our estimate of 12.2% for PDS is higher than that of earlier cross‐sectional German studies on dysthymia using categorical diagnosis (e.g., 2.5% in 65+ [2] and 5.1% in 85+ [23]), but aligns more closely with longitudinal European findings using dimensional diagnosis: Netherlands 6.5% [53], for England 6.9% [42] and overall European countries of 7.3% [22]. Taking only majorPDS (2.5%) or dysthymia estimates (e.g., 2.5% by [2]) as a proxy for PDD likely underestimates its true burden [10]. Our estimate of 12.2% for PDS is concerning, in view of the known increased likelihood of greater health service utilization among those with PDS, but nevertheless lacking adequate treatment initiation [54]. Our findings support that DS remain frequent in later‐life. Whether cases represent first onset in late‐life or recurrence of DS could not be determined in our study. DS increase in late‐life [55], in particular among the oldest old, which is an age at which depression onset is also strongly associated with increased risk for Alzheimer's dementia [56].

Surprisingly, there was no increase in DS observed for the waves during and after the COVID‐19 pandemic. This stands in stark contrast to international literature [57, 58, 59] where prevalence estimates doubled, with specific subgroups (e.g., low socioeconomic status, poor health) at particular risk [60]. German literature also shows an increase [61], with one study utilizing DEAS data as well [62]. There may be multiple possibilities for this difference. Beside methodology (e.g., we used weights) and sampling (e.g., we have 60+ year olds living in private households), it is possible that the COVID‐19 pandemic and restrictions had a particular impact on individuals who were already at risk (e.g., widowed, lonely, poor health) and above the depression threshold [63]. Increases in depressive symptom scores in already affected individuals may therefore not be reflected in the prevalence and incidence shown. In addition, the study mentioned above [62] used data from the DEAS covid‐survey conducted in the summer 2020. The data used in our analyses comes from the fall and winter of 2020, at a time when people were better placed to adapt to (restrictions of) the pandemic (e.g., improved use of technology for social connection, vaccinations were on the horizon). While older adults showed higher anxiety regarding a covid infection [64], evidence suggests that they showed higher resilience and higher levels of coping than younger adults [65, 66]. Younger adults cope more through social contacts and may have been at greater risk during social restrictions [67].

While approximately one‐third of major depression cases become persistent in the general population [18], our results support single studies [14, 15] indicating higher chronification rates of up to 50%–in our data around 40%. Our results further highlight the role of minor depression as a key risk factor for major depression, with 3 times higher transition rates to major DS from minor DS compared to those from no DS. Also, transition from major to minor DS were very common–indicating high residual symptoms and few full remissions after major depressive episodes. Similar transition patterns were reported for China [40], where transition intensity from severe DS to mild DS, instead of directly to no DS, was 11 times higher.

Time‐constant determinants (e.g., female gender, lower education and predispositions [68]) were not examined in regression analysis in our study, though our findings revealed statistically significant time‐varying determinants. Widowed individuals are a particularly vulnerable group, with studies showing increased levels of DS up to 5 years after the life event [69]. One previous study tracked DS over 14 years and found similar short‐ and long‐term effects of widowhood on DS in both men and women [70]. However, women are more likely to become widowed–often earlier in less favorable conditions–partly explaining common unfavorable depression trajectories. Further, poor physical functioning, sleeping problems and loneliness were linked to increases in DS, consistent with past studies [11, 68]. While sleeping problems are a consistent risk factor for the development [71] and persistence [72] of late‐life depression, it should be noted that they can also be a symptom of depression. The theory of selection, optimization and compensation [73] proposes that DS arise when individuals are unable to adjust to losses associated with aging. This might explain why the number of physical illnesses was not associated with DS, but lower physical functioning was, indicating a lack of coping strategies. Sleeping problems are particularly common in late‐life depression, with residual difficulties often persisting after remission and even serving as a predictor for relapse [11]. Loneliness, rather than network size, seems to be a key psychosocial risk factor. Negative association of BMI with depressive symptoms may reflect unintentional weight loss, a key symptom of frailty. Frailty is shown to be as risk factor for depression and depression also heightens risk for incident frailty [74]. The results are largely consistent with the literature on psychological resilience in older adults, a factor i.e. reversely associated with depressive symptoms [75]. In particular, lifestyle factors (e.g., continuing physical activity), social support and preserving a purpose appear to be predictive of maintaining mental health despite age‐related losses [76].

### 4.1 Strengths and Limitations

This study draws on representative data from the carefully conducted DEAS study. Longitudinal data comes from the six most recent waves spanning a total of 15 years. DS were assessed using an established depression screening tool, and applying comprehensive DSM‐V definition of PDD is a rarity in literature to date. Prevalence was reported in detail by age, gender and wave. FE regression allowed robust estimation of time‐varying predictors, markedly reducing the challenge of unobserved heterogeneity. A wide array of determinants was included in the regression model.

However, a few limitations exist. While the response rate of 27.1% for 2014 might appear low by international standards, it is comparable to high quality cross‐sectional health surveys from Germany [2, 32, 33] and international studies with similar design and method [77]. Response rate in subsequent waves (around 65%) [34] showed good adherence of the panel samples. Analyses of representativeness show only a small selection bias (less participation in large cities, among women, and among the oldest old) [31]. Weights were used to adjust for these factors. Still, we cannot fully dismiss the possibility that particular groups (e.g., very severely impaired individuals) are underrepresented in our analysis. The estimates are based on a screening tool and may overestimate the prevalence due to a higher rate of false positives. While the CES‐D cut‐off for major depression is validated [39], there is no consensus threshold for minor depression [12, 78]. Thus, classification remains debated. Our definition of PDD as showing corresponding symptoms in two consecutive waves might include individuals with temporary full remission, potentially inflating prevalence. However, this approach follows other studies on persistent DS using longitudinal health survey data [22, 42, 53], and our analyses suggest that full remissions are less common among older adults. Incomplete remission cases are still classifying as PDD by DSM‐V [17]. Our results reflect longitudinal associations and reverse causality cannot be excluded. There is a lack of data on psychiatric history and current treatment. Among other things, this means that it is not possible to say whether DS have reoccurred in old age or are of late‐onset.

### 4.2 Future Research

Distinguishing more explicitly between new depression onset and recurrence of depression in late‐life might be promising in identifying different risk groups. Furthermore, epidemiological studies including institutionalized individuals in e.g., nursing homes would provide deeper understanding. The DEAS sample did not include these groups. As they are known to have higher rates of late‐life depression [11, 79] this might potentially underestimate true burden in older adults. Finally, further potential determinants (e.g., alcohol consumption, cognitive functioning), should be investigated. This study did not include them due to inconsistent measurement across waves.

## 5 Conclusion

Depressive episodic and persistent symptoms are common in older adults in Germany. Minor DS frequently precedes major DS and both frequently take a persistent course. Early targeted interventions could be informed by modifiable determinants. This may help to allocate scarce mental health resources effectively to prevent progression and persistence of late‐life depression.

## Funding

The authors have nothing to report.

## Ethics Statement

The study was conducted in accordance with the Declaration of Helsinki. An ethical statement for the DEAS study was not necessary, because the criteria for requiring an ethical statement were not met (risk for the respondents, lack of information about the aims of the study, examination of patients). The German Center of Gerontology, who is responsible for the DEAS study, did not apply for an ethics vote, based on the recommendation of a standing council of the DEAS, who decided that an ethics vote would not be necessary.

## Conflicts of Interest

The authors declare no conflicts of interest.

## Supporting information


Supporting Information S1

## Data Availability

All data are available from the German Center of Gerontology. For further details (application for data use): https://www.dza.de/en/research/fdz/access‐to‐data/application.
