# Supplementary material for: Persistence and Determinants of Late‐Life Depression: Results of the Nationally Representative Longitudinal German Aging Survey 2008 to 2023
Source: Int J Geriatr Psychiatry. 2025 Dec 17;40(12):e70181. doi: 10.1002/gps.70181 (PMC12710116; doi:10.1002/gps.70181)
Supplement: Supplementary file 1 — Supporting Information S1 [file GPS-40-e70181-s001.docx]

**Supplementary Material to Article**

“Prevalence, Incidence, Persistence, and Determinants of Late-Life Depression: Results of the Nationally Representative Longitudinal German Ageing Survey 2008 to 2023”

**Content**

[**Table 1.** Post-hoc comparisons of sociodemographic information in 2014 2](#_Toc210812282)

[**Table 2.** Prevalence and incidence information in 2014 by age group 5](#_Toc210812283)

[**Table 3.** Prevalence and incidence information by wave 6](#_Toc210812284)

[**Table 4.** Depression transitions Men 7](#_Toc210812285)

[**Table 5.** Depression transitions Women 7](#_Toc210812286)

[**Table 6.** Depression transitions 60-69 year olds 8](#_Toc210812287)

[**Table 7.** Depression transitions 70-79 year olds 8](#_Toc210812288)

[**Table 8.** Depression transitions 80+ year olds 9](#_Toc210812289)

[**Table 9.** Fixed Effects Regression for men 10](#_Toc210812290)

[**Table 10.** Fixed Effects Regression for women 11](#_Toc210812291)

[**Table 11.** Fixed Effects Regression for 60-69 year olds 12](#_Toc210812292)

[**Table 12.** Fixed Effects Regression for 70-79 year olds 13](#_Toc210812293)

[**Table 13.** Fixed Effects Regression for 80+ year olds 14](#_Toc210812294)

# **Table 1.** Post-hoc comparisons of sociodemographic information in 2014

|  | DS Stage | | | Comparisons | | |
| --- | --- | --- | --- | --- | --- | --- |
|  | no DS | minor DS | major DS | No DS vs. minor DS | No DS vs. major DS | Minor DS vs. major DS |
| N | 4,981 (76.5%) | 1,119 (17.2%) | 415 (6.4%) |  |  |  |
| Age | 71.1 (7.1) | 72.4 (7.7) | 72.2 (7.7) | <0.001 | 0.002 | 0.685 |
| Age group |  |  |  |  |  |  |
| 60-69 years | 2,130 (42.8%) | 407 (36.4%) | 158 (38.1%) | <0.001 | <0.001 | 0.341 |
| 70-79 years | 2,205 (44.3%) | 495 (44.2%) | 167 (40.2%) |  |  |  |
| 80+ years | 646 (13.0%) | 217 (19.4%) | 90 (21.7%) |  |  |  |
| Gender |  |  |  |  |  |  |
| Men | 2,764 (55.5%) | 510 (45.6%) | 159 (38.3%) | <0.001 | <0.001 | 0.011 |
| Women | 2,217 (44.5%) | 609 (54.4%) | 256 (61.7%) |  |  |  |
| Level of Education (ISCED) |  |  |  |  |  |  |
| low | 392 (7.9%) | 146 (13.0%) | 84 (20.2%) | <0.001 | <0.001 | <0.001 |
| medium | 2,484 (49.9%) | 613 (54.8%) | 230 (55.4%) |  |  |  |
| high | 2,103 (42.2%) | 360 (32.2%) | 101 (24.3%) |  |  |  |
| Marital Status |  |  |  |  |  |  |
| married | 3,572 (71.9%) | 712 (63.8%) | 224 (54.5%) | <0.001 | <0.001 | 0.008 |
| married, living separately | 59 (1.2%) | 16 (1.4%) | 11 (2.7%) |  |  |  |
| divorced | 420 (8.4%) | 107 (9.6%) | 41 (10.0%) |  |  |  |
| widowed | 736 (14.8%) | 237 (21.2%) | 111 (27.0%) |  |  |  |
| single | 184 (3.7%) | 44 (3.9%) | 24 (5.8%) |  |  |  |
| Number of physical disease (self-report) [0-11] | 2.7 (1.8) | 3.8 (2.0) | 4.1 (2.1) | <0.001 | <0.001 | 0.033 |
| Physical functioning [0-100] | 82.5 (20.6) | 62.3 (28.9) | 49.8 (32.5) | <0.001 | <0.001 | <0.001 |
| Loneliness [1-4] | 1.7 (0.5) | 2.0 (0.6) | 2.2 (0.6) | <0.001 | <0.001 | <0.001 |
| Network size [0-9+] | 5.0 (2.8) | 4.6 (2.8) | 4.3 (2.6) | <0.001 | <0.001 | 0.049 |
| Sleep quality |  |  |  |  |  |  |
| Very good | 1,027 (25.7%) | 83 (9.9%) | 15 (5.2%) | <0.001 | <0.001 | <0.001 |
| Good | 2,282 (57.1%) | 379 (45.1%) | 90 (31.5%) |  |  |  |
| Fairly bad | 630 (15.8%) | 317 (37.7%) | 134 (46.9%) |  |  |  |
| Very bad | 58 (1.5%) | 61 (7.3%) | 47 (16.4%) |  |  |  |
| Body-Mass-Index | 27.0 (4.3) | 27.3 (4.8) | 27.6 (5.3) | 0.017 | 0.004 | 0.296 |
| Physical Activity |  |  |  |  |  |  |
| Never | 1,463 (29.4%) | 478 (42.7%) | 225 (54.3%) | <0.001 | <0.001 | <0.001 |
| Less often | 513 (10.3%) | 114 (10.2%) | 22 (5.3%) |  |  |  |
| 1 to 3 times a month | 321 (6.4%) | 66 (5.9%) | 12 (2.9%) |  |  |  |
| Once a week | 856 (17.2%) | 182 (16.3%) | 55 (13.3%) |  |  |  |
| Several times per week | 1,317 (26.5%) | 202 (18.1%) | 68 (16.4%) |  |  |  |
| Daily | 509 (10.2%) | 77 (6.9%) | 32 (7.7%) |  |  |  |
| **Abbreviations.** DS: Depressive symptoms; ISCED: International Standard Classification of Education.  **Note**. DS assessed using the 15-item German version of the Center for Epidemiologic Studies Depression Scale (CES-D): no DS (0-9), minor DS (10-17) and major DS (18-45). Physical functioning measured using the subscale “Physical functioning” of the Short Form Health Survey 36. Loneliness measured with the widely used De Jong Gierveld scale. | | | | | | |

# **Table 2.** Prevalence and incidence information in 2014 by age group

|  | Age group | | | | | |
| --- | --- | --- | --- | --- | --- | --- |
|  | 60-69 years | | 70-79 years | | 80+ years | |
| DS Stage |  |  |  |  |  |  |
| no DS | 2,130 | 79.3%  [77.5%-81.0%] | 2,205 | 76.6%  [74.3%-78.8%] | 646 | 62.6%  [56.6%-68.2%] |
| minor DS | 407 | 14.7%  [13.3%-16.3%] | 495 | 17.3%  [15.3%-19.4%] | 217 | 25.8%  [20.6%-31.7%] |
| major DS | 158 | 5.9%  [5.0%-7.1%] | 167 | 6.1%  [5.1%-7.4%] | 90 | 11.6%  [8.2%-16.1%] |
| Minor DS Incidence | 90 | 9.2%  [7.3%-11.4%] | 101 | 10.3%  [7.9%-13.3%] | 56 | 10.1%  [7,1%-14.1%] |
| Major DS Incidence | 37 | 3.9%  [2.6%-5.7%] | 42 | 3.6%  [2.6%-5.1%] | 24 | 6.9%  [3.7%-12.4%] |
| PDS | 87 | 10.0%  [7.8%-12.6%] | 112 | 11.5%  [9.2%-14.2%] | 61 | 23.4%  [15.5%-33.7%] |
| majorPDS | 15 | 2.2%  [1.2%-4.0%] | 22 | 3.4%  [2.1%-5.4%] | 8 | 6.5%  [1.9%-20.4%] |
| PDS Incidence | 25 | 2.9%  [1.8%-4.5%] | 40 | 3.4%  [2.4%-4.8%] | 32 | 15.4%  [9.2%-24.6%] |
| majorPDS Incidence | 4 | 0.6%  [0.2%-1.9%] | 10 | 1.1%  [0.5%-2.1%] | 3 | 3.5%  [0.6%-17.0%] |
| **Abbreviations.** DS: Depressive symptoms; PDS: Persistent depressive symptoms.  **Note.** DS assessed using the 15-item German version of the Center for Epidemiologic Studies Depression Scale (CES-D): no DS (0-9), minor DS (10-17) and major DS (18-45); minor DS incidence (score ≥ 10 and < 18 in current wave and < 10 in previous wave); major DS incidence (score ≥ 18 and < 18 in previous wave); PDS (score ≥ 10) and majorPDS (score ≥ 18) in two consecutive waves. | | | | | | |

#

# **Table 3.** Prevalence and incidence information by wave

|  | wave | | | | | | | | | | | |
| --- | --- | --- | --- | --- | --- | --- | --- | --- | --- | --- | --- | --- |
|  | 1 | | 2 | | 3 | | 4 | | 5 | | 6 | |
| DS Stage |  |  |  |  |  |  |  |  |  |  |  |  |
| no DS | 3,590 | 76.7% [75.2%-78.1%] | 2,486 | 74.4% [72.0%-76.7%] | 4,981 | 75.3% [73.7%-76.9%] | 3,650 | 75.0% [72.9%-77.1%] | 3,243 | 75.0% [71.7%-78.1%] | 3,138 | 75.9% [73.2%-78.4%] |
| minor DS | 777 | 16.9% [15.6%-18.1%] | 513 | 17.4% [15.5%-19.5%] | 1,119 | 17.7% [16.3%-19.2%] | 826 | 18.1% [16.3%-20.1%] | 729 | 17.0% [14.9%-19.4%] | 601 | 17.0% [14.8%-19.3%] |
| major DS | 298 | 6.5% [5.7%-7.3%] | 210 | 8.2% [6.7%-9.9%] | 415 | 7.0% [6.1%-8.0%] | 274 | 6.9% [5.8%-8.1%] | 248 | 7.9% [5.6%-11.2%] | 199 | 7.1% [5.6%-9.0%] |
| Minor DS Incidence |  |  | 233 | 9.3% [7.7%-11.2%] | 247 | 9.8% [8.4%-11.5%] | 438 | 9.5% [8.3%-10.8%] | 358 | 8.9% [7.7%-10.4%] | 280 | 12.4% [8.2%-18.5%] |
| Major DS Incidence |  |  | 115 | 5.8% [4.2%-8.0%] | 103 | 4.5% [3.4%-5.9%] | 184 | 4.9% [4.0%-5.9%] | 155 | 5.0% [3.3%-7.3%] | 127 | 3.6% [2.7%-4.8%] |
| PDS |  |  | 296 | 13.1% [11.4%-15.0%] | 260 | 13.7% [11.3%-16.6%] | 499 | 13.1% [11.6%-14.8%] | 406 | 14.6% [12.3%-17.2%] | 361 | 11.0% [8.8%-13.6%] |
| majorPDS |  |  | 48 | 2.6% [1.8%-3.8%] | 45 | 3.7% [2.1%-6.4%] | 76 | 2.2% [1.7%-2.8%] | 55 | 2.5% [1.8%-3.5%] | 47 | 2.8% [1.5%-5.2%] |
| PDS Incidence |  |  |  |  | 97 | 5.2% [3.8%-7.1%] | 93 | 4.4% [3.5%-5.7%] | 136 | 3.9% [3.2%-4.8%] | 119 | 3.1% [2.4%-3.9%] |
| majorPDS Incidence |  |  |  |  | 17 | 1.3% [0.6%-3.0%] | 18 | 0.7% [0.4%-1.1%] | 25 | 0.9% [0.5%-1.5%] | 15 | 0.5% [0.3%-0.9%] |
| **Abbreviations**. DS: Depressive symptoms; PDS: Persistent depressive symptoms.  **Note**. DS assessed using the 15-item German version of the Center for Epidemiologic Studies Depression Scale (CES-D): no DS (0-9), minor DS (10-17) and major DS (18-45); minor DS incidence (score ≥ 10 and < 10 in previous wave); major DS incidence (score ≥ 10 and < 18 in current wave and < 10 in previous wave); PDS (score ≥ 10) and majorPDS (score ≥ 18) in two consecutive waves. | | | | | | | | | | | | |

# **Table 4.** Depression transitions Men

|  | DS Stage | | | | | |
| --- | --- | --- | --- | --- | --- | --- |
|  | no DS | | minor DS | | major DS | |
| Consecutive DS Stage |  |  |  |  |  |  |
| no DS | 4,121 | 88.2% [87.0%-89.3%] | 389 | 53.8% [48.9%-58.6%] | 57 | 33.2% [24.5%-43.2%] |
| minor DS | 454 | 9.4% [8.4%-10.5%] | 248 | 36.5% [31.9%-41.3%] | 66 | 39.7% [30.6%-49.5%] |
| major DS | 94 | 2.4% [1.8%-3.2%] | 71 | 9.7% [7.4%-12.7%] | 35 | 27.1% [19.0%-37.2%] |
| **Abbreviations**. DS: Depressive symptoms.  **Note**. DS assessed using the 15-item German version of the Center for Epidemiologic Studies Depression Scale (CES-D): no DS (0-9), minor DS (10-17) and major DS (18-45). | | | | | | |

# **Table 5.** Depression transitions Women

|  | DS Stage | | | | | |
| --- | --- | --- | --- | --- | --- | --- |
|  | no DS | | minor DS | | major DS | |
| Consecutive DS Stage |  |  |  |  |  |  |
| no DS | 3,297 | 79.4% [77.1%-81.4%] | 487 | 49.4% [44.9%-53.9%] | 101 | 28.4% [18.1%-41.6%] |
| minor DS | 568 | 16.0% [14.1%-18.0%] | 342 | 37.8% [33.5%-42.3%] | 123 | 29.2% [22.4%-37.0%] |
| major DS | 154 | 4.7% [3.7%-6.0%] | 111 | 12.9% [9.9%-16.6%] | 122 | 42.4% [32.7%-52.7%] |
| **Abbreviations**. DS: Depressive symptoms.  **Note**. DS assessed using the 15-item German version of the Center for Epidemiologic Studies Depression Scale (CES-D): no DS (0-9), minor DS (10-17) and major DS (18-45). | | | | | | |

# **Table 6.** Depression transitions 60-69 year olds

|  | DS Stage | | | | | |
| --- | --- | --- | --- | --- | --- | --- |
|  | no DS | | minor DS | | major DS | |
| Consecutive DS Stage |  |  |  |  |  |  |
| no DS | 3,484 | 87.7% [86.4%-88.9%] | 392 | 55.5% [51.0%-60.0%] | 85 | 30.5% [24.4%-37.3%] |
| minor DS | 379 | 9.4% [8.4%-10.6%] | 224 | 35.5% [31.3%-40.0%] | 83 | 35.3% [28.4%-42.9%] |
| major DS | 93 | 2.9% [2.3%-3.7%] | 64 | 9.0% [6.8%-11.7%] | 71 | 34.2% [27.3%-41.9%] |
| **Abbreviations**. DS: Depressive symptoms.  **Note**. DS assessed using the 15-item German version of the Center for Epidemiologic Studies Depression Scale (CES-D): no DS (0-9), minor DS (10-17) and major DS (18-45). | | | | | | |

# **Table 7.** Depression transitions 70-79 year olds

|  | DS Stage | | | | | |
| --- | --- | --- | --- | --- | --- | --- |
|  | no DS | | minor DS | | major DS | |
| Consecutive DS Stage |  |  |  |  |  |  |
| no DS | 3,010 | 82.5% [80.5%-84.4%] | 343 | 48.7% [43.2%-54.2%] | 48 | 19.1% [12.8%-27.6%] |
| minor DS | 426 | 13.3% [11.8%-15.0%] | 245 | 36.3% [31.1%-41.7%] | 79 | 32.6% [23.9%-42.7%] |
| major DS | 106 | 4.2% [3.0%-5.7%] | 82 | 15.0% [10.8%-20.5%] | 61 | 48.3% [36.2%-60.5%] |
| **Abbreviations**. DS: Depressive symptoms.  **Note**. DS assessed using the 15-item German version of the Center for Epidemiologic Studies Depression Scale (CES-D): no DS (0-9), minor DS (10-17) and major DS (18-45). | | | | | | |

# **Table 8.** Depression transitions 80+ year olds

|  | DS Stage | | | | | |
| --- | --- | --- | --- | --- | --- | --- |
|  | no DS | | minor DS | | major DS | |
| Consecutive DS Stage |  |  |  |  |  |  |
| no DS | 924 | 75.2% [69.9%-79.8%] | 141 | 47.1% [38.8%-55.6%] | 25 | 47.3% [21.5%-74.7%] |
| minor DS | 217 | 20.6% [16.2%-25.8%] | 121 | 42.4% [34.2%-51.0%] | 27 | 23.8% [11.6%-42.7%] |
| major DS | 49 | 4.2% [2.6%-6.6%] | 36 | 10.5% [7.0%-15.4%] | 25 | 28.8% [13.9%-50.5%] |
| **Abbreviations**. DS: Depressive symptoms.  **Note**. DS assessed using the 15-item German version of the Center for Epidemiologic Studies Depression Scale (CES-D): no DS (0-9), minor DS (10-17) and major DS (18-45). | | | | | | |

# **Table 9.** Fixed Effects Regression for men

|  | DS | |
| --- | --- | --- |
| Age | -0.01 |  |
|  | (0.01) |  |
| Marital Status |  |  |
| married, living separately | -0.67 |  |
|  | (1.22) |  |
| divorced | 0.34 |  |
|  | (0.75) |  |
| widowed | 0.85 | * |
|  | (0.39) |  |
| single | 1.68 |  |
|  | (1.15) |  |
| Number of physical disease (self-report) [0-11] | 0.07 |  |
|  | (0.04) |  |
| Physical functioning [0-100] | -0.07 | ** |
|  | (0.00) |  |
| Loneliness [1-4] | 0.78 | ** |
|  | (0.16) |  |
| Network size [0-9+] | 0.00 |  |
|  | (0.02) |  |
| Sleep quality |  |  |
| Good | 0.51 | ** |
|  | (0.16) |  |
| Fairly bad | 2.19 | ** |
|  | (0.24) |  |
| Very bad | 3.60 | ** |
|  | (0.57) |  |
| Body-Mass-Index | -0.10 | ** |
|  | (0.03) |  |
| Physical Activity |  |  |
| Less often | -0.03 |  |
|  | (0.16) |  |
| 1 to 3 times a month | -0.21 |  |
|  | (0.20) |  |
| Once a week | -0.02 |  |
|  | (0.17) |  |
| Several times per week | -0.13 |  |
|  | (0.15) |  |
| Daily | 0.05 |  |
|  | (0.24) |  |
| Intercept | 12.26 | ** |
|  | (1.36) |  |
| Number of observations | 11200 |  |
| Number of groups | 4756 |  |
| R-squared | 0.12 |  |
| ** p<.01, * p<.05.  Abbreviations. DS: Depressive symptoms.  Note. DS assessed using the 15-item German version of the Center for Epidemiologic Studies Depression Scale (CES-D). Physical functioning measured using the subscale “Physical functioning” of the Short Form Health Survey 36. Loneliness measured with the widely used De Jong Gierveld scale. | | |

# **Table 10.** Fixed Effects Regression for women

|  | DS | |
| --- | --- | --- |
| Age | 0.03 | * |
|  | (0.02) |  |
| Marital Status |  |  |
| married, living separately | 1.27 |  |
|  | (0.94) |  |
| divorced | -0.92 |  |
|  | (1.23) |  |
| widowed | 0.44 |  |
|  | (0.34) |  |
| single | -1.81 |  |
|  | (1.71) |  |
| Number of physical disease (self-report) [0-11] | 0.00 |  |
|  | (0.05) |  |
| Physical functioning [0-100] | -0.06 | ** |
|  | (0.00) |  |
| Loneliness [1-4] | 1.24 | ** |
|  | (0.19) |  |
| Network size [0-9+] | -0.03 |  |
|  | (0.03) |  |
| Sleep quality |  |  |
| Good | 1.17 | ** |
|  | (0.18) |  |
| Fairly bad | 3.23 | ** |
|  | (0.24) |  |
| Very bad | 5.12 | ** |
|  | (0.48) |  |
| Body-Mass-Index | -0.07 | * |
|  | (0.03) |  |
| Physical Activity |  |  |
| Less often | 0.27 |  |
|  | (0.23) |  |
| 1 to 3 times a month | -0.44 |  |
|  | (0.31) |  |
| Once a week | -0.42 |  |
|  | (0.25) |  |
| Several times per week | -0.76 | ** |
|  | (0.23) |  |
| Daily | -0.59 | * |
|  | (0.29) |  |
| Intercept | 7.69 | ** |
|  | (1.49) |  |
| Number of observations | 10246 |  |
| Number of groups | 4351 |  |
| R-squared | 0.11 |  |
| ** p<.01, * p<.05.  Abbreviations. DS: Depressive symptoms.  Note. DS assessed using the 15-item German version of the Center for Epidemiologic Studies Depression Scale (CES-D). Physical functioning measured using the subscale “Physical functioning” of the Short Form Health Survey 36. Loneliness measured with the widely used De Jong Gierveld scale. | | |

# **Table 11.** Fixed Effects Regression for 60-69 year olds

|  | DS | |
| --- | --- | --- |
| Age | -0.09 | ** |
|  | (0.02) |  |
| Marital Status |  |  |
| married, living separately | 1.09 |  |
|  | (1.10) |  |
| divorced | 0.28 |  |
|  | (0.86) |  |
| widowed | 1.69 | * |
|  | (0.66) |  |
| single | -0.84 |  |
|  | (1.80) |  |
| Number of physical disease (self-report) [0-11] | -0.03 |  |
|  | (0.06) |  |
| Physical functioning [0-100] | -0.07 | ** |
|  | (0.01) |  |
| Loneliness [1-4] | 0.15 |  |
|  | (0.19) |  |
| Network size [0-9+] | 0.00 |  |
|  | (0.03) |  |
| Sleep quality |  |  |
| Good | 0.76 | ** |
|  | (0.16) |  |
| Fairly bad | 2.44 | ** |
|  | (0.28) |  |
| Very bad | 4.60 | ** |
|  | (0.71) |  |
| Body-Mass-Index | -0.00 |  |
|  | (0.04) |  |
| Physical Activity |  |  |
| Less often | 0.09 |  |
|  | (0.25) |  |
| 1 to 3 times a month | 0.19 |  |
|  | (0.31) |  |
| Once a week | -0.24 |  |
|  | (0.24) |  |
| Several times per week | -0.35 |  |
|  | (0.24) |  |
| Daily | -0.29 |  |
|  | (0.35) |  |
| Intercept | 16.22 | ** |
|  | (1.81) |  |
| Number of observations | 9047 |  |
| Number of groups | 5142 |  |
| R-squared | 0.07 |  |
| ** p<.01, * p<.05.  Abbreviations. DS: Depressive symptoms.  Note. DS assessed using the 15-item German version of the Center for Epidemiologic Studies Depression Scale (CES-D). Physical functioning measured using the subscale “Physical functioning” of the Short Form Health Survey 36. Loneliness measured with the widely used De Jong Gierveld scale. | | |

# **Table 12.** Fixed Effects Regression for 70-79 year olds

|  | DS | |
| --- | --- | --- |
| Age | 0.07 | ** |
|  | (0.02) |  |
| Marital Status |  |  |
| married, living separately | 0.00 |  |
|  | (1.22) |  |
| divorced | 0.14 |  |
|  | (1.55) |  |
| widowed | 1.18 |  |
|  | (0.61) |  |
| single | -1.48 |  |
|  | (4.41) |  |
| Number of physical disease (self-report) [0-11] | 0.03 |  |
|  | (0.06) |  |
| Physical functioning [0-100] | -0.06 | ** |
|  | (0.01) |  |
| Loneliness [1-4] | 0.76 | ** |
|  | (0.25) |  |
| Network size [0-9+] | -0.04 |  |
|  | (0.03) |  |
| Sleep quality |  |  |
| Good | 0.58 | ** |
|  | (0.22) |  |
| Fairly bad | 2.62 | ** |
|  | (0.33) |  |
| Very bad | 4.28 | ** |
|  | (0.80) |  |
| Body-Mass-Index | -0.12 | * |
|  | (0.05) |  |
| Physical Activity |  |  |
| Less often | -0.05 |  |
|  | (0.25) |  |
| 1 to 3 times a month | -0.45 |  |
|  | (0.35) |  |
| Once a week | -0.44 |  |
|  | (0.23) |  |
| Several times per week | -0.51 | * |
|  | (0.25) |  |
| Daily | -0.14 |  |
|  | (0.33) |  |
| Intercept | 7.07 | ** |
|  | (2.44) |  |
| Number of observations | 8738 |  |
| Number of groups | 5004 |  |
| R-squared | 0.10 |  |
| ** p<.01, * p<.05.  Abbreviations. DS: Depressive symptoms.  Note. DS assessed using the 15-item German version of the Center for Epidemiologic Studies Depression Scale (CES-D). Physical functioning measured using the subscale “Physical functioning” of the Short Form Health Survey 36. Loneliness measured with the widely used De Jong Gierveld scale. | | |

# **Table 13.** Fixed Effects Regression for 80+ year olds

|  | DS | |
| --- | --- | --- |
| Age | 0.14 | ** |
|  | (0.05) |  |
| Marital Status |  |  |
| married, living separately | 1.78 |  |
|  | (5.54) |  |
| divorced | -4.37 |  |
|  | (2.60) |  |
| widowed | 0.87 |  |
|  | (0.57) |  |
| single | 3.12 |  |
|  | (2.74) |  |
| Number of physical disease (self-report) [0-11] | 0.05 |  |
|  | (0.10) |  |
| Physical functioning [0-100] | -0.05 | ** |
|  | (0.01) |  |
| Loneliness [1-4] | 1.54 | ** |
|  | (0.41) |  |
| Network size [0-9+] | 0.03 |  |
|  | (0.06) |  |
| Sleep quality |  |  |
| Good | 0.58 |  |
|  | (0.35) |  |
| Fairly bad | 2.24 | ** |
|  | (0.53) |  |
| Very bad | 4.75 | ** |
|  | (1.06) |  |
| Body-Mass-Index | -0.11 |  |
|  | (0.08) |  |
| Physical Activity |  |  |
| Less often | -0.11 |  |
|  | (0.50) |  |
| 1 to 3 times a month | -1.40 | * |
|  | (0.56) |  |
| Once a week | -0.06 |  |
|  | (0.44) |  |
| Several times per week | -0.76 | * |
|  | (0.36) |  |
| Daily | -0.29 |  |
|  | (0.40) |  |
| Intercept | -1.72 |  |
|  | (5.26) |  |
| Number of observations | 3661 |  |
| Number of groups | 2260 |  |
| R-squared | 0.17 |  |
| ** p<.01, * p<.05.  Abbreviations. DS: Depressive symptoms.  Note. DS assessed using the 15-item German version of the Center for Epidemiologic Studies Depression Scale (CES-D). Physical functioning measured using the subscale “Physical functioning” of the Short Form Health Survey 36. Loneliness measured with the widely used De Jong Gierveld scale. | | |
